# A review of individual multidisciplinary team roles in alcohol and other drugs

**DOI:** 10.3389/fmed.2025.1512703

**Published:** 2025-08-29

**Authors:** Xuan Ye, Leighlan Phillips, Caity Morrison, Jo Zhuang, Brit Chapman, Hannah Portogallo, Michael Hodge, Jo Linden, Antigone Branchflower, Craig Harvey, Robert M. Lundin

**Affiliations:** ^1^Faculty of Medicine, Nursing and Health Sciences, Monash University, Melbourne, VIC, Australia; ^2^Mental Health Services, Alcohol and Other Drugs Integrated Treatment Team, Mildura Base Public Hospital, Mildura, VIC, Australia; ^3^Academic Regional and Rural Addictions Network (ARRAN), Geelong, VIC, Australia; ^4^Mental Health Drugs and Alcohol Services, Barwon Health, Geelong, VIC, Australia; ^5^Institute for Mental and Physical Health and Clinical Translation (IMPACT), Deakin University, Geelong, VIC, Australia; ^6^Harm Reduction Victoria (HRVIC), Melbourne, VIC, Australia

**Keywords:** alcohol and other drugs, addiction, peer work, social work, nursing, pharmacy, occupational therapy, harm reduction

## Abstract

Alcohol and Other Drugs (AOD) usage remains high globally and in Australia and results in a high amount of morbidity and mortality in our community. The Australian AOD workforce comprises a highly diverse range of healthcare professionals. Since medical doctors and AOD roles without specific credentials have already been covered in the literature, this review summarizes on the other direct patient facing roles in the AOD multidisciplinary team. We focused on harm reduction workers, peer workers, social workers, pharmacists, occupational therapists, psychologists, nurses and nurse practitioners. The findings of this review show that previous non-specific roles such as peer and harm reduction workers are developing into their distinct disciplines with their own specific training. Experienced clinicians in their field such as nurse practitioners and senior pharmacists are given increasingly more scope of practice that aligns with medical practitioners. However, there is still limited research to some established disciplines such as the role occupational therapy and social work. Future research should focus on how these roles can be fully utilized in the AOD multidisciplinary team.

## Introduction

Alcohol and Other Drugs (AOD) usage remains high globally and in Australia and continues to contribute to significant morbidity and mortality in our society. In Australia, where deaths involving stimulants have increased from 0.8 per 100,000 population in 2013 to 1.8 per 100,000 population in 2022, opioids remain the most common at 4.0 per 100,000 population (1). Cannabis was reported to be the leading drug-causing drug use disorders in half of the countries surveyed by United Nation Office on Drugs and Crime (UNODC), including Australia (2, 3).

Looking at the impact of alcohol, it remains a large contributor to mortality and morbidity. Alcohol can be attributed to 4.7% of all deaths in 2019 (4). Alcohol damages a range of body organs such as the liver, the brain and the heart causing cardiomyopathies (5). In Australia, there were 1,742 alcohol-induced deaths, which represents 0.91% of all deaths in 2022. While the percentage is relatively low compared to the global numbers, it is a 9.1% increase from 2021 (6). Alcohol is also attributed to 59% of all drug-related Australian hospitalization in 2021–2022 (7).

Out of 1,280 publicly funded AOD treatment services in Australia in 2022–2023, 69% were non-government organizations (8). It has changed from 2012–2013 when only 56% of them were non-government (8). The location of these services has remained consistent 57% in 2022–2023 is in major cities (8, 9). Non-government workers have been found to be younger, less experienced and with lower formal qualifications. However, they report higher job satisfaction and meaning from their work (10).

The Australian AOD workforce comprises a highly diverse range of healthcare and non-healthcare professionals. In a 2019–2020 survey, the National Center for Education and Training for Addiction (NCETA) recorded 35 occupations in the Australian AOD workforce, ranging from medical practitioners, pharmacists and counselors to service managers (11). The last national survey was done in 2005 and recognized significantly fewer occupations, with only doctors, social workers, psychologists, nurses, counselors/therapists and general AOD workers who often do not have specific professional qualifications (12).

Lundin and Hill (13) recently examined the doctor's role within the addiction space, highlighting how no single specialty holds full responsibility for it. Instead, the responsibilities are shared by general medical specialties (inpatient withdrawals and consequences of substance misuse), primary care (outpatient withdrawals and pharmacotherapy), and psychiatry (addiction and psychological consequences). Core competencies such as pharmacotherapy and other prescribing, diagnosing relevant disorders, and guiding treatment plans have also been described (14).

Since medical doctors and AOD roles without specific qualifications have previously been described in the literature, this mini review focuses on the other roles and disciplines within the AOD multidisciplinary team. It aims to highlight the skills and unique functions each group provides while also attempting to highlight any significant literature gaps to guide future research. Two different search methods were used. Through the Ovid platform, the databases PsychINFO and Emcare were searched using a combination of discipline specific subject headings and keywords with adjustment of the search terms to suit each database. We also used the research specific AI search engine Elicit (15) to explore the Semantic Scholar Corpus database for suitable articles. The engine was only used to discover suitable papers, with no AI generated content used in our review.

## Harm reduction

Harm reduction can be broadly described as a range of pragmatic strategies, programs and interventions that seek to minimize the harms associated with substance use (16). Harm reduction practitioners come from diverse personal and professional backgrounds (17). They are united by a shared set of values, a comprehensive understanding of the drivers of drug use, and a goal to help others enhance any aspects of life, including spiritual, physical, and mental wellbeing (18).

Harm reduction values safer practices to avoid the negative consequences of drug use. Core components of harm reduction work include the provision of safer injecting and safer sex products, drug testing/checking, drug safety education, medication-assisted treatment for opioid dependence, overdose prevention, and reducing the transmission of blood-borne viruses (16, 19). Harm reduction practitioners recognize the broad spectrum of drug use beyond abstinence and diagnoses. This includes the role of pleasure in the drug use experience, along with drugs acting as a functional mediator of life. Harm reduction work considers this spectrum when providing any care, adopting a person-centered approach, and aiming to provide nuance in any service provision (18).

The stigmatization of people who use drugs when entering the healthcare system, including within AOD services is evident and a known barrier to seeking treatment (20–22). A focus of harm reduction work is to challenge stigma, and provide advocacy, at an individual, organizational, and systemic level.

Harm reduction advocacy efforts are an essential part of the discipline. Such advocacy is achieved through meaningful co-design of services, strengthening the entire workforce through appropriate person-first language, and advancing policy aims to include evidence-based health interventions such as drug consumption services (23, 24).

The values and principles that underpin harm reduction are grounded in social inclusion. Harm reduction recognizes the inherent dignity of every person and challenges the notion of drug use as an act of deviance. With its roots in compassion, harm reduction workers aim to foster inclusivity (25).

## Peer work

In the AOD space, a peer worker is defined as someone who uses their lived or living experience of substance use to inform their work (26). While other workers in the field may have personal experience of drug use, they differ from peer workers in that their role is not defined by that lived or living experience, and they may choose to disclose, or not (27). A recent milestone in Australia in the development of the peer workforce was reached in 2017 with the funding and establishment of the Reducing Harmful Drug Use through Peer Led Networks (RHDUPLN) initiative, which sought to utilize peer workers' connections to local drug using communities to address and provide solutions to overdose risk and other drug-related harms (28).

Initially peer workers worked on the periphery and weren't a consideration for inclusion as part of core multidisciplinary teams, however they are increasingly recognized and valued, as demonstrated by the growing number of formal roles being created across the AOD sector (27, 29–31).

Peer-led interventions and strategies for safer drug use are evaluated as highly effective for achieving improved health-based outcomes for people who use drugs (PWUD), such as lower transmission of HIV between people who inject drugs (PWID) and prevention of overdose (31–33). Other common tasks performed include safe facilitation of referrals, accompanying clients to appointments, and advocating on their behalf when requested to (32, 34).

Recent studies demonstrate that peers derive much meaning and motivation from the work, citing feelings of pride, purpose and belonging, resulting in high levels of compassion satisfaction and act as a powerful protective factors against any negative impacts, even in the face of overdose crisis and the ongoing deaths of community and loved ones (35, 36).

## Social work

Despite the limited emphasis on addiction training in social work education, social workers have historically been, and continue to be, key providers of services for individuals who experience substance use disorders (37).

A social work lens frames problematic substance uses as a matter of social care, viewing addiction as a response to deep-seated social isolation and disconnection rather than an individual's moral failing. Some theories used to conceptualize addiction include dislocation theory and fragmented intimacy theory. These perspectives emphasize a socio-ecological and contextual understanding of addiction, utilizing a biopsychosocial and ecological framework for assessment that considers family dynamics, neighborhood and community influences (38).

Dislocation theory suggests that interventions based solely on individuals are often ineffective as they fail to tackle the underlying causes of addiction. Treatment is more likely to succeed when orientated toward restoring psychosocial integration and preventing dislocation (38).

As social workers spend the majority of time in direct contact with clients, those without a specialized AOD role contribute by (1) engaging with the topic of substance use, (2) motivating people to consider changing their substance-using behavior and supporting them to do so and (3) supporting people to make and maintain changes—which includes not only the individual themselves but also the systems around them (39).

The role of a social worker who specializes in addiction will be guided by their agency's requirements and approach to substance use support and intervention and influenced by local and national government strategic priorities (39). This could include providing holistic assessment, support and advocacy, crisis intervention, case management, outreach, counseling, therapy, family systems work and psychosocial rehabilitation across the public, private or non-government organizational domains.

## Pharmacy

The role of pharmacists in the AOD space is broad and expanding. Firstly, community pharmacists are valuable members of the opioid substitution treatment team and play a role in the education, dispensing and administration of methadone or buprenorphine (40). Similarly, pharmacists also provide naloxone in the community (41). However, some pharmacists in New Zealand have reported that they felt the current broader treatment team needed to understand their role in the team (40). In Australian areas where there is a shortage of medical practitioners for opioid substitution treatment, pharmacists have been allowed to co-prescribe opioid substitution treatment with an appropriately qualified medical practitioner (42). Improved patient continuity of care and improved patient monitoring and convenience were regarded as the main benefits from the patient and pharmacists' perspective (42). As a follow-up, Cheetham et al. (43) found that prescribers of opioid substitution treatment (suitably qualified doctors and nurse practitioners) supported greater involvement from pharmacists in co-prescribing provided that the pharmacists had suitable credentials.

In the United States, pharmacists have also been reported to be involved in drug courts and to be successfully part of inpatient addiction triage teams as consultants for improving education on AOD medications (44, 45). With the rise of vaping in Australia, pharmacists also have a crucial role in educating the public on nicotine vaping products that are available in pharmacies as a harm reduction tool for quitting smoking (46).

## Occupational therapy

King et al. (47) described occupational therapists' more specialized role in the community mental health setting. This paper highlighted the potential impact and underutilized role that OTs can play in providing enhanced holistic care to clients and general community occupational therapists (OT), taking a more integrated approach and recognizing the role they can play in screening mental health concerns. Unfortunately, there was no mention of AOD screening as part of or separate from the proposed mental health intervention.

In general practice, effective screening, with brief interventions, has been shown to improve clinical outcomes and demonstrate economic viability (48). The chronic nature of health conditions treated by general community OTs means that the rates of AOD concerns and their impact on overall health outcomes will be intensified. Whilst needing intervention, AOD concerns are not usually the client's primary presenting concern. OTs are well placed to identify AOD concerns; their community role allows insights into clients' overall functioning and factors impacting their participation in daily life (49).

## Nursing and nurse practitioners

Nurses assume many roles in drug and alcohol services. Not only are they one of the primary professions providing care in drug and alcohol rehabilitation services, and specialist drug and alcohol services they also play a role in identification and screening in primary care, hospital and general practice settings. Nurses commencing their career in the workforce, having completed an undergraduate degree, usually have very little (or sometimes nil) training in identifying and working with consumers who have drug and alcohol issues (50). Smyth et al. (51) surveyed nurses in a regional health district and found that nurses were only minimally confident in assessment referral protocols, when these are essential tasks in treating hospital patients with AOD use disorders. Goodhew et al. (50) co-produced an Australian undergraduate nursing AOD subject with experts by lived experience, expert AOD nurses and nurse educators. They subsequently delivered the subject in 2021 and 2022 and found that the subject improved students' understanding of trauma informed care and harm reduction (52).

Within the AOD workforce there are also Nurse Practitioners (NPs) who are highly skilled registered nurses who, through advanced tertiary education, expand their scope of practice to include clinical activities such as diagnosing, prescribing pharmacological treatments, admitting or discharging patients and referring to specialists. In Australia, the NPs standards of care provide a minimum standard that NPs must adhere to and spans four domains: clinical, education, research and leadership (53).

In the AOD settings, particularly in rural areas, NPs provide clinical services in managing substance use disorders and associated health comorbidities, depending on their individual training and scope of practice (54). Their unique nursing background enables them to adopt a holistic, patient-centered approach that integrates physical and mental health care.

In multidisciplinary teams (MDTs) in AOD services, the role of NPs can include prescribing Medication-Assisted Treatment for Opioid Dependence (MATOD), managing withdrawal syndromes, and screening, diagnosing, and treating blood-borne viruses (BBVs), sexually transmitted infections (STIs) and common co-morbid chronic conditions. Additionally, NPs provide leadership, education, and contribute to research in these settings (54).

Despite their potential, NPs in AOD settings face significant barriers. These include limited organizational support, few opportunities for advanced education, a shortage of NPs positions, and sometimes resistance from colleagues (55). Funding disparities and lack of adequate access to national reimbursement schemes like the Medicare Benefits Schedule (MBS) can further restrict the scope of NPs practice (56). Addressing these barriers is essential to fully leverage the capacity of NPs in enhancing access to care and improving health outcomes for underserved populations (56).

## Psychology

Psychologists bring specialized expertise in assessment and behavior change, positioning them as leaders in addiction treatment (57). They also conduct screenings for substance use disorders (SUDs) through validated tools and informal questioning, identifying individuals in need of support (58). Key approaches include screening, brief Intervention, and referral to treatment, motivational interviewing (MI), cognitive-behavioral therapy (CBT), and relapse prevention strategies (57–65). In psychotherapy, psychologists typically take a preventative approach by addressing risk factors and fostering protective factors against addiction (58).

Although SUD-specific training continues to develop in some post-graduate programs (59), < 40% of clinical psychology programs focus on addiction, leading to a lack of specialized training for a large population of psychologists working with this cohort (66). Many psychologists working in the public health space, often work with opioid use disorders and lack the necessary training to address such issues (67).

Psychologists can significantly impact the stigma associated with SUDs through their therapeutic work, promoting resilience and reducing internalized stigma (63). As stigma complicates treatment and perpetuates health inequalities, it is crucial for psychologists to enhance their training and research efforts in this area (63, 68). This includes developing competencies in SUD diagnosis and treatment and advocating for policy changes to improve integrated care for individuals facing concurrent mental health and/or substance use disorders (59).

## Discussion

The Alcohol and Other Drugs field has many discipline-agnostic roles, such as counselors or “AOD workers” where there is no specific pre-determined training or qualification (11, 12). This review examined the specific roles and functions that harm reduction workers, peer workers, nurses, psychologists, social workers and occupational therapists form within a multidisciplinary team.

### Limited research into some established healthcare disciplines

There is limited literature on the role of social workers and occupational therapists within the AOD domain. Although both roles appear to frequently be employed in the AOD space, there is limited description of how the unique skill set of these roles are adapted to addiction (69). Although there are some descriptions of how the roles can employ generic AOD functions, like substance use screening and brief interventions, these are limited descriptions of how discipline specific skills such as functional and sensory assessments can be utilized (38, 48). Whilst all healthcare professionals should be trained to provide adequate screening, brief intervention and appropriate referral to specialist AOD services, these disciplines should be supported to further impact the field (39).

### A strong evidence base in nursing

The more recent evolution of nurse practitioner roles has allowed these highly trained nurses to diagnose and prescribe medications for substance use disorders, functions that have previously been limited to medical practitioners (54). It is also promising that there is an active effort in improving AOD education within the nursing curriculum (50, 52). But there is a concerning description in the literature of stigma and resistance toward AOD-specific clinicians and their patients from their general colleagues (70, 71).

### Previously non-specific roles developing into distinct disciplines

By comparison, there is a rapidly growing evidence base within both peer and harm reduction work. Due to their common origin, there is a considerable degree of overlap between these roles in the literature but the development of discipline specific training, core values and functions means these previously non-specific AOD roles are developing their own governance and networks (27, 29). Interestingly, there is an emerging overlap between these disciplines and traditional healthcare roles, this is evident in how the peer and harm reduction values overlap with established social work values and in how harm reduction services focus on reducing spread of infectious diseases (16, 18, 25).

### Future work

This review has identified that there are concerning gaps in how established healthcare disciplines like social work and occupational therapy lack research into their specific function within the AOD-field. Future research should focus on determining how these roles can be fully utilized within an AOD multidisciplinary team (MDT). More research is also needed in determining how these roles differ between urban and rural areas (72, 73). Ideally, these gaps should be used as a template for systematic reviews for each field.
